# Associations of odor identification with CSF biomarkers for Alzheimer’s disease and central olfactory system volumes in subjective cognitive impairment, mild cognitive impairment, and Alzheimer’s disease

**DOI:** 10.1002/alz70862_109879

**Published:** 2025-12-23

**Authors:** Javier Oltra, Zuzana Wallin Ištvánfyová, Grégoria Kalpouzos, Ingrid Ekström, Göran Hagman, Miia Kivipelto, Erika J Laukka

**Affiliations:** ^1^ Aging Research Center, Department of Neurobiology, Care Sciences and Society, Karolinska Institutet and Stockholm University, Stockholm, Stockholm Sweden; ^2^ Division of Clinical Geriatrics, Center for Alzheimer Research, Department of Neurobiology, Care Sciences and Society, Karolinska Institutet, Stockholm, Stockholm Sweden; ^3^ Theme Inflammation and Aging, Karolinska University Hospital, Stockholm, Stockholm Sweden; ^4^ Institute of Public Health and Clinical Nutrition, University of Eastern Finland, Kuopio, Kuopio Finland; ^5^ Ageing Epidemiology Research Unit, School of Public Health, Imperial College London, London, England UK; ^6^ Stockholm Gerontology Research Center, Stockholm, Stockholm Sweden

## Abstract

**Background:**

Olfactory deficits predict future dementia and Alzheimer’s Disease (AD). It is crucial to unravel the mechanisms of olfactory dysfunction in the pre‐dementia stages to understand their potential as early markers. We aimed to examine the associations of AD‐related CSF biomarkers and central olfactory system volumes with odor identification (OID) in subjective cognitive impairment (SCI), mild cognitive impairment (MCI), and AD.

**Method:**

Individuals with SCI (*n* = 154; *M*_age_=58.5, *SD_age_* = 5.7; %_females_=64.9), MCI (*n* = 51; *M*_age_=61.6, *SD_age_* = 5.0; %_females_=49.0), and AD (*n* = 31; *M*_age_=58.7, *SD_age_* = 5.0; %_females_=54.8) were recruited from the Karolinska University Hospital Memory Clinic, Solna, Sweden. We examined within‐group associations of OID, assessed with the Sniffin' Sticks test (number of correct identifications, range 0 to 16), with CSF biomarkers (42 amino acid form of amyloid‐β [Aβ42], tau phosphorylated at threonine 181 [*p*‐tau181], and neurofilament light chain [NfL]), and central olfactory system volumes extracted using cNeuro cMRI software (Combinostics Oy; left hippocampus, amygdala, left parahippocampal gyrus, entorhinal cortex, orbitofrontal cortex, insula, and left caudate). Data were analyzed using one‐tailed partial correlations for all groups (controlled for age, sex, and education) and multiple linear regressions applying stepwise model selection for SCI and MCI. The alpha level was set at 0.05.

**Result:**

SCI (*M* = 14.1, *SD* = 2.2) outperformed MCI (*M* = 12.7, *SD* = 3.5) and AD groups (*M* = 12.8, *SD* = 2.8) in OID. In SCI, Aβ42 (*r* = 0.136) and orbitofrontal volume (*r* = 0.194) were associated with OID. In MCI, hippocampal (*r* = 0.347), parahippocampal (*r* = 0.274), and caudate volumes (*r* = 0.343) were associated with OID. In AD, hippocampal volume (*r* = 0.557) was associated with OID. Aβ42 and orbitofrontal volume were significant predictors of OID in the linear regression model in SCI (adjusted‐*R^2^* =  0.102); whereas NfL, entorhinal, and insula volumes were significant predictors of OID in MCI (adjusted‐*R^2^* = 0.497).

**Conclusion:**

Olfactory deficits were associated with amyloid CSF levels and lower orbitofrontal volume in individuals with SCI, suggesting that amyloid deposition and orbitofrontal degeneration may play a role early in the disease spectrum. In MCI and AD, degeneration of medial temporal lobe and subcortical structures may contribute to these deficits.

